# Impact of Ambient and Elevated [CO_2_] in Low Light Levels on Growth, Physiology and Nutrient Uptake of Tropical Perennial Legume Cover Crops

**DOI:** 10.3390/plants10020193

**Published:** 2021-01-20

**Authors:** Virupax C. Baligar, Marshall K. Elson, Zhenli He, Yuncong Li, Arlicelio de Q. Paiva, Alex-Alan F. Almeida, Dario Ahnert

**Affiliations:** 1 USDA-ARS-Beltsville Agricultural Research Center, Beltsville, MD 20705, USA; marshall.elson@usda.gov; 2 Indian River Research and Education Center, Department of Soil and Water Sciences, IFAS, University of Florida, Fort Pierce, FL 34945, USA; zhe@ufl.edu; 3 Tropical Research and Education Center, Department of Soil and Water Sciences, IFAS, University of Florida, Homestead, FL 33031, USA; yunli@ufl.edu; 4 Department of Agricultural and Environmental Science, State University of Santa Cruz, Ilhéus, BA 45650-000, Brazil; arli@uesc.br; 5 Department of Biological Science, State University of Santa Cruz, Ilhéus, BA 45650-000, Brazil; alexalan@uesc.br (A.-A.F.A.); darioa@uesc.br (D.A.)

**Keywords:** nutrient uptake, influx and transport, nutrient and water use efficiency, net photosynthesis

## Abstract

At early stages of establishment of tropical plantation crops, inclusion of legume cover crops could reduce soil degradation due to erosion and nutrient leaching. As understory plants these cover crops receive limited irradiance and can be subjected to elevated CO_2_ at ground level. A glasshouse experiment was undertaken to assess the effects of ambient (450 µmol mol^−1^) and elevated (700 µmol mol^−1^) levels of [CO_2_] on growth, physiological changes and nutrient uptake of six perennial legume cover crops (Perennial Peanut, Ea-Ea, Mucuna, Pigeon pea, Lab lab, Cowpea) under low levels of photosynthetic photon flux density (PPFD; 100, 200, and 400 µmol m^−2^ s^−1^). Overall, total and root dry biomass, total root length, specific leaf area, and relative growth rates were significantly influenced by levels of [CO_2_] and PPFD and cover crop species. With few exceptions, all the cover crops showed significant effects of [CO_2_], PPFD, and species on net photosynthesis (*P_N_*) and its components, such as stomatal conductance (*gs*) internal CO_2_ conc. (*Ci*)*,* and transpiration (*E*). Increasing [CO_2_], from 450 to 700 μmol mol^−1^ and increasing PPFD from 100 to 400 μmol ּm^−2^ ּs^−1^ increased *P_N_*. Overall, the levels of [CO_2_], PPFD and species significantly affected total water use efficiency (*WUE_TOTAL_*), instantaneous water use efficiency (*WUE_INST_*) and intrinsic water use efficiency (*WUE_INTR_*). With some exceptions, increasing levels of [CO_2_] and PPFD increased all the *WUE* parameters. Interspecific differences were observed with respect to macro-micro nutrient uptake and use efficiency. With a few exceptions, increasing levels of [CO_2_] from 450 to 700 μmol mol^−1^ and PPFD from 100 to 400 μmol m^−2^ s^−1^ increased nutrient use efficiency (NUE) of all nutrients by cover crop species.

## 1. Introduction

In the tropics, plantation crops such as coffee, cacao, tea and banana are often established with wide row spacing on recently cleared, sloping land. Loss of the vegetative cover causes soil degradation due to massive soil erosion and leaching of nutrients. Fast growing cover crops in early establishment of tropical plantation crops have been shown to control soil erosion, nutrient leaching, and weed infestations, improve organic matter and nutrient content, conserve soil moisture, and enhance beneficial soil physical properties [1,2,3,4].

Soil incorporated residues from cover crops improve soil organic matter and this in turn improves soil fertility as well as its physical, chemical and biological properties, thereby restoring soil productivity [4,5,6,7]. Growth and development of cover crops are influenced by environmental variables such as light intensity, temperature, rainfall and soil fertility [4,8]. In plantation crops, adequate light at the canopy level is a problem for growth and development of cover crops. As the tree crops and shade trees mature, understory plants such as cover crops, suffer from inadequate levels of photosynthetic photon flux density (PPFD) for their growth. In tropical regions, incoming PPFD is around 1800 µmol m^−2^ s^−1^ [9], but understory plants may receive only 4–10% of incoming PPFD [10,11]. In agroforestry-based plantations, cover crops receive full sunlight during early stages of plantation crop establishment but as the upperstory plantation trees grow, incoming PPFD reaching the cover crop canopy is reduced. Cover crops have varying degrees of tolerance to low light intensity and in many instances will not survive longer than a few years because they are suppressed by reduced light quality [2,12,13]. Low PPFD at the crop’s canopy level reduces growth, development and nutrient use efficiency of cover crops [14,15,16,17]. The ability of understory cover crops to survive in plantation crops depends largely on the intensity and quality of light reaching their canopies [4,8]. Limited information is available on inter- and intra-specific differences in tropical perennial cover crops for tolerance to shade [18,19,20]. Shading is known to reduce yields of many tropical legumes and heavy shade can affect their survivability in plantation crops [21,22]. Very limited published evidence exists in areas of tropical perennial legume cover crops response to low to adequate light intensities [14,15,16]. However, the ability of many tropical legume cover crop species to grow at low light intensity is unknown.

Cover crops that tolerate reduced PPFD have greater potential to survive longer and to reduce soil degradation, improve soil C sequestration, and control weed infestations in plantation crops. Interspecific differences in nine perennial legume cover crop species have been reported for growth and macro-micronutrient uptake and use efficiency and increasing PPFD from 200 to 400 µmol m^−2^ s^−1^ increased all the growth and nutrient uptake traits [14,15]. Reducing PPFD from 1000 to 50 µmol m^−2^ s^−1^ in five tropical perennial legume cover crops (calopo, jack bean, mucuna, white lead tree and perennial peanut) reduced photosynthesis to less than 10% of the higher light level [16]. Similarly, in four Crotalaria cover crop species increasing PPFD from 50 to 1500 µmol m^−2^ s^−1^ increased photosynthesis by 21-fold [23]. Very limited information is available on the growth, physiology, and nutrition of various cover crops under varying light intensities.

Light quality at the crop canopy under artificial shade is very different from the quality of light at canopy levels of understory plants in field conditions. Light reaching understory plants in the field could be low in PPFD, with a low red/far red (R/FR) ratio and relatively enriched with green and red light, depending on nature and density of leaf cover of the upper story trees [24,25]. Therefore, plant response to artificial shade might be different than plants grown under shade of upper story shade trees because of differences in light quality.

Atmospheric carbon dioxide concentration [CO_2_] is expected to double by the end of this century from the current level of 400 µmol mol^−1^ [26,27]. Increased litter decomposition in plantation crops also contributes to higher [CO_2_] at the ground levels. Even under adequate light, nutrients and water status, elevated [CO_2_] can contribute to increased biomass and physiological parameters such as net photosynthesis (*P_N_*), water use efficiency (*WUE*) and nutrient use efficiency (*NUE*) by plants [16,17,23,28,29,30]. Increased atmospheric CO_2_ leads to higher *P_N_* and creates additional demand for nutrients by the plants as long as light quality and intensity are adequate. Baligar et al. Ref. [16] evaluated independent short-term effects of [CO_2_] and PPFD on several tropical perennial legumes. In these studies, increasing [CO_2_] from 250 to 700 μmol mol^−1^ doubled *P_N_*. Increasing PPFD from 50 to 1000 μmol m^−2^ s^−1^ increased *P_N_* more than 10% of that at the lower levels. In another study with crotalaria species, Baligar et al. Ref. [23] reported that increasing external [CO_2_] from 100 to 1000 µmol mol^−1^ increased *P_N_* by 4.7-fold and increasing PPFD from 50 to 1500 µmol m^−2^ s^−1^ increased *P_N_* by 21-fold. Recently, Baligar et al. Ref. [17] reported the effects of increasing [CO_2_] from 400 to 700 µmol mol^−1^ at varying levels of low PPFD (100, 250, and 450 µmol m^−2^ s^−1^) on five tropical legume cover crop species (Calopo/frisolla, Jack bean, Brazilian lucerne, Leucena, and Mucuna). Overall, in these legume species, increasing [CO_2_] and PPFD significantly increased all the growth parameters, water use efficiency and nutrient uptake efficiency. Interspecific differences in growth, physiological and NUE traits in tropical perennial legume cover crops have been reported for varying light intensities [14,15,16,23], shade tolerance [18,19,20,31], and ambient and elevated levels of [CO_2_] [16,17,23,30].

The objective of this research was to assess the impact of ambient (400 µmol mol^−1^) and elevated (700 µmol mol^−1^) levels of [CO_2_] on growth, physiological and nutrient uptake of six tropical perennial legume cover crops grown at several low levels of PPFD (100, 250 and 450 ± 50 µmol m^−2^ s^−1^). Information gained from this study will be useful for identification of cover crop species that can tolerate reduced PPFD (increased shade) and maintain longer longevity as the PPFD levels reduce as the canopy of upperstory trees increases with time. From this study, more needed information will be gained on how perennial legume cover crops used as understory plants in tropical plantations respond to increasing atmospheric [CO_2_] in reducing or increasing PPFD.

## 2. Results and Discussion

### 2.1. [CO_2_] and PPFD Effects on Growth Parameters

With few exceptions, overall shoot, root and leaf parameters, and relative growth rate (RGR), were significantly influenced by levels of [CO_2_], PPFD and crop species and their interactions (Table 1). With the exception of specific leaf area (SLA), increasing [CO_2_] from 450 to 700 μmol mol^−1^ and PPFD from 100 to 400 µmol m^−2^ s^−1^ increased all the growth parameters and *RGR*. Baligar et al. [17,25] showed that increasing [CO_2_] from 400 to 700 μmol mol^−1^ increased growth traits of many tropical perennial legume crop species. Doubling of atmospheric [CO_2_] has been shown to increase plant biomass by almost 40% [32]. However, the growth response in different plant species to increasing [CO_2_] is not consistent [16,17,23,28,29,32].

Reductions in yield among tropical legume cover crops under low light intensities has been reported [13,21,22,33]. Baligar et al. Refs. [15,17,30] reported that shoot, leaf and root growth parameters of tropical legume cover crops increased significantly by increasing PPFD from 100 to 450 µmol m^−2^ s^−1^. Baligar et al. Refs. [17,30] reported that in several species of tropical perennial legume cover crops, increasing [CO_2_] from 400 to 700 µmol mol^−1^ and PPFD from 100 to 450 µmol m^−2^ s^−1^ increased growth parameters (total root and shoot biomass, root/shoot ratio, stem height, relative growth rates), water and macro-micro nutrient use efficiency. In the current study, overall root/shoot ratio decreased with decreases in light intensity, indicating that low light intensity is detrimental, especially to shoot growth. Mucuna recorded the highest shoot dry weight, root length and leaf area; Ea-Ea recorded the lowest shoot and root weight, stem height, root length and leaf area. Mucuna had a high leaf area and high *P_N_*, thereby resulting in higher dry matter accumulations than the other cover crop species tested. Moss [34] reported that plants with larger leaf area have greater potential for dry matter accumulation than those with smaller leaf area. Baligar et al. Ref. [14] reported that perennial tropical legume cover crops such as Sunn hemp, Cowpea and Lab-lab with larger leaf areas accumulated higher dry biomass in shoots and roots than cover crops with smaller leaf areas such as Joint vetch, Hairy indigo and Crotalaria. Irrespective of levels of [CO_2_] and PPFD, Mucuna recorded the longest root lengths and Ea-Ea recorded the shortest root length. Such a long root system might help the plant to absorb more water and nutrients by exploring a larger soil volume, and thus these cover crops could be suitable for infertile soils of tropical plantation crops. Baligar et al. Ref. [17] also reported the largest root systems in Mucuna as compared to many other cover crops irrespective of levels of [CO_2_] and PPFD. The beneficial effects of cover crops grown under plantation crops such as cacao are observed during the first 3–4 years of establishment, as the cacao and the associated shade trees grow and heavy canopy is formed, the effectiveness of the cover crop diminishes, because of lack of sufficient light [13,22,33].

Significant interaction effects between increasing [CO_2_] and cover crop species were observed on total and root dry wt, total root length, leaf area, specific leaf area and RGR; however, a significant interaction of [CO_2_] and PPFD was only observed for total root wt. (Table 1). With the exception of stem height, highly significant interaction effects of PPFD and cover crop species were observed with other growth parameters and RGR. Higher levels of PPFD have significant effects on growth and sustainability of these cover crops; therefore, in tropical plantation crops, to enhance longevity of cover crops effectiveness, canopy management of upper story tree crops is vital.

### 2.2. [CO_2_] and PPFD Effects on Physiological Parameters

With few exceptions, all of the cover crops showed significant effects of [CO_2_], PPFD, species and their interactions on *P_N_* and its components (*gs* and *Ci*), SPAD index and nutrient assimilation ratio (NAR) (Table 2). Overall, increasing [CO_2_] from 450 to 700 μmol mol^−1^ increased *P_N_*, *Ci* and NAR. Increasing PPFD from 100 to 400 μmol m^−2^ s^−1^ increased *P_N_*, *gs* and NAR, and decreased *Ci*. Baligar et al. Ref. [16] evaluated independent short-term effects of PPFD and [CO_2_] on photosynthesis of perennial peanut (*Arachis pintoi*), calopo (*Calopogonium mucunoides*), jack bean (*Canavalia ensiformis*), leucena (*Leucaena leucocephala*) and mucuna (*Mucuna pruriens*). In all these legume species, increasing external [CO_2_] from 250 to 700 cm^3^ m^−3^ doubled *P_N_* and reducing PPFD from 1000 to 480 μmol m^−2^ s^−1^ reduced *P_N_* to less than 10%. Baligar et al. [23] in a short-term study reported that in four Crotalaria species (*C. breviflora*, *C. mucronata, C. ochroleuca, C. spectabilis*), increasing PPFD from 50 to 1000 μmol m^−2^ s^−1^ increased instantaneous *P_N_* by 21 fold, increased instantaneous *gs* by 2.3 fold, and decreased instantaneous *Ci* by 3.9 times. Increasing [CO_2_] from 100 to 1000 cm^3^ m^−3^ increased instantaneous *P_N_* by 4.7 fold, decreased instantaneous *gs* by 1.3 times, and increased instantaneous *Ci* by 28 fold.

The SPAD index was unaffected by levels of [CO_2_] and PPFD but cover crops species showed significant differences for SPAD. Izaguirre-Mayoral et al. [33] reported significant reductions in leaf chlorophyll in 24 native legume species of Venezuela grown at 75% reduction of the incident sunlight (480 μmol m^−2^ s^−1^). Inter-specific differences for shade tolerance [18,19,20,31] for varying light intensities in cover crops have been reported [14,15,16,23].

Highly significant interaction effects between increasing [CO_2_] and cover crop species were observed for *P_N_*, *gs*, and NAR (Table 2). Highly significant interaction effects of PPFD and cover crop species were observed for SPAD, *P_N_*, *gs*, water flux, and NAR. Cover crop species adapted in this study significantly responded to increasing PPFD. To maintain adequate levels of photosynthesis and NAR of understory cover crops in tropical plantation crops, it is essential to manage the canopy of upper story tree species to increase light levels (PPFD) at cover crop canopy levels.

### 2.3. [CO_2_] and PPFD Effects on Water Flux and Water Use Efficiency

Overall, the levels of [CO*_2_*], PPFD and species significantly affected total water use efficiency (*WUE_TOTAL_*), instantaneous water use efficiency (*WUE_INST_*) and intrinsic water use efficiency (*WUE_INTR_)*; however, [CO_2_] and PPFD had no effect on Water Flux (Vo) (Table 2). Increasing levels of [CO_2_] and PPFD increased *WUE_INST_* and *WUE_INTR_*. *WUE_TOTAL_* increased with increasing [CO_2_] and reduced with increasing PPFD. In other crops, it has been reported that relationships between *WUE_TOTAL_* and *WUE_INST_* may be either positive or negative [35]. Inter-specific differences were observed in water use efficiency parameters. WUE was significantly influenced by interaction effects between cover crop species and increasing [CO_2_] and PPFD.

### 2.4. [CO_2_] and PPFD Effects on Macro- and Micronutrient Uptake

#### 2.4.1. Nutrient Concentrations and Uptake

Concentrations of N and P in all of these cover crops were slightly higher than the reported concentrations in the literature, and all other essential nutrients were at adequate levels (Table 3) [14,15,36,37].

With few exceptions, overall concentrations of macro- and micronutrients were significantly influenced by levels of [CO_2_], PPFD and cover crop species. Inter-specific differences were observed with respect to nutrient concentrations. Increasing [CO_2_] from 450 to 700 μmol mol^−1^ decreased concentrations of N, K and Mn, but increased concentrations of other nutrients. Increasing PPFD from 100 to 400 μmol m^−2^ s^−1^ increased only the concentrations of N, K and Ca. Overall concentrations were in the order of N > K >P = Ca > Mg for macronutrients and Mn > Fe > Zn > Cu for micronutrients. In other tropical legume cover crop species, Baligar et al. Refs. [14,30] reported a similar pattern of nutrient concentrations.

Macro- and micro-nutrient uptake were significantly influenced by levels of [CO_2_], PPFD and cover crop species (Table 4). Increasing levels of [CO_2_] from 450 to 700 μmol mol^−1^ and PPFD from 450 to 700 μmol m^−2^ s^−1^ increased the uptake of all the macro- and micronutrients.

The effect of shading on nutritive value is often negative [19]. The differential effects of varying levels of PPFD on nutrient uptake by perennial cover crop legumes have been reported [14,15,17,30]. In greenhouse conditions with varying levels of shade (18 to 100% of daylight), Wong [38] reported changes in mineral composition of Joint Vetch, Calopo, Centro, Ea-Ea, Tropical Kudzu and Brazilian Lucerne. In this study, the mean P, Ca, Mg and K content in all the legumes increased significantly with increasing shade (low levels of PPFD). Baligar et al. Refs. [15,30] reported significant responses to increasing levels of PPFD from 100 to 450 μmol m^−2^ s^−1^ for nutrient uptake in cover crop legume species. Significant variability in nutrient uptake among various cover crop species is associated with different growth habits, the amount of dry matter accumulated in the shoot and the specific demand of the plant for any particular nutrient [15,39]. Concentrations of K, Mg, Cu and Fe and uptake of Ca, Mg, Fe, Mn, and Zn were significantly influenced by interaction effects between increasing [CO_2_] and cover crop species (Table 3 and Table 4). Concentrations and uptake of all the macro-micro nutrients were significantly influenced by interaction effects between increasing PPFD and cover crop species. Across all crop species, [CO_2_], and PPFD levels, uptake of nutrients was in the order of N > K > P > Ca > Mg for macro nutrients and Mn > Fe > Zn >Cu for micronutrients. Fageria et al. Ref. [40] and Baligar et al. Refs. [14,15,30] have reported similar trends in nutrient uptake by legume crops.

#### 2.4.2. Nutrient Influx (IN) and Transport (TR)

Nutrient influx (IN) of all nutrients was significantly influenced but varied by species (Table 5). With the exception of Mn, IN for all other nutrients increased significantly with increasing PPFD from 100 to 400 μmol m^−2^ s^−1^. Levels of [CO_2_] significantly affected IN for only N and Mg. Baligar et al. Ref. [17] reported that in other tropical legumes, with a few exceptions, levels of [CO_2_] and PPFD had no significant effects on IN of nutrients. In an earlier study by Baligar et al. Ref. [14] with nine tropical legume cover crops, IN for macro- and micronutrients was significantly affected by species but PPFD had no significant effect on the IN of nutrients.

Transport (TR) of macro- and micronutrients was significantly influenced by species and PPFD (Table 6). However, levels of [CO_2_] significantly affected TR of N, Mg, Cu, and Fe only. In many other legume cover crops species, Baligar et al. Refs. [14,15,30] reported similar effects of varying levels of [CO_2_] and PPFD. In the current study, with few exceptions, overall increasing levels of [CO_2_] and PPFD increased the TR for all the nutrients.

Influx and transport of P, Mg, and Fe were significantly influenced by interaction effects between increasing [CO_2_] and cover crop species (Table 5 and Table 6). However, influx of N, P, Mg, Fe and Mn were only significantly influenced by interaction effects between PPFD and cover crop species. Interaction effects between increasing [CO_2_] and cover crop species significantly influenced transport of P, K, Mg, Fe, and Cu. However, with the exception of transport of K, all other macro and micro nutrient transport was highly significantly influenced by interaction effects between PPFD and cover crop species.

#### 2.4.3. Nutrient Use Efficiency (NUE)

Nutrient use efficiency in plants is profoundly influenced by levels of available nutrients in soil (supply) and genetic and physiological components (demand) of plants [41,42]. Light levels at the canopy and ambient [CO_2_] levels have a great influence on plant demands for nutrients [14,17,30,32,38,42]. However, information is limited on the influence of various light levels and [CO_2_] levels on NUE of tropical legume cover crops [14,15,17,30]. Overall, the NUE of all the macro- and micronutrients was significantly influenced by cover crop species (Table 7). With few exceptions, macro- and micronutrient use efficiency was significantly influenced by levels of [CO_2_] and PPFD. In the cover crop species tested, increasing levels of [CO_2_] from 450 to 700 μmol mol^−1^ and PPFD from 100 to 400 μmol m^−2^ s^−1^ increased the NUE of all nutrients. Perennial Peanut was the most efficient in the use of N, Cu and Mn. Ea-Ea was most efficient in NUE for P and Fe. Overall, crop species showed inter-specific differences for NUE of macro- and micronutrients. Interspecific variations for macro- and micronutrient use efficiency are well documented in legume cover crops [14,15,30,42,43]. With the exception of NUE for Ca, Fe and Zn, the NUEs for all other macro-micro nutrients were significantly influenced by interaction effects between increasing [CO_2_] and cover crop species (Table 7). However, with the exception of NUE for Cu, the NUEs for all other macro-micro nutrients were highly significantly influenced by interaction effects between PPFD and cover crop species.

## 3. Materials and Methods 

### 3.1. Perennial Legume Cover Crops

Six perennial legume cover crops were used in this study: Perennial Peanut (*Arachis pintoi* Krapov. & W.C. Greg), Ea-Ea (*Desmodium heterocarpon* (L.) DC subsp. *ovalifolium* (Prain) Ohashi), Mucuna (*Mucuna pruriens* (L.) DC var *utilis* (Wall ex Wight) L.H. Bailey), Pigeon pea (*Cajanus cajan* (L.) Millsp.), Lab-lab (*Lablab purpureus* (L.) Sweet) and Cowpea (*Vigna unguiculata* (L.) Walp.). Growth habits, strengths and limitations of cover crops used are given in Table 8.

Table 8 lists the growth habits, strengths and limitations of these cover crops. Advantages and disadvantages of these cover crop species have been extensively cited by Duke [44]; Cook [45]; Faridah Hanum and van der Maesen [46]; Cook et al. [47]; Fageria et al. [6]; Baligar and Fageria [8]; and Fageria et al. [4]. Perennial peanut is a stoloniferous, perennial herb, native to South America, and can produce 5–10 t ha^−1^ yr^−1^ of dry matter (DM). It is somewhat tolerant of shade and can tolerate high levels of soil Mn and Al [45]. Ea-Ea is a non-climbing perennial vine, native to Southeast Asia, and can produce 1–7 t ha^−1^ yr^−1^ DM and fix 45–200 kg ha^−1^ yr^−1^ N [47]. Mucuna is a vigorous, twining herb, native to southern China, which is used as a cover crop and can produce 2–12 t ha^−1^ yr^−1^ DM and fix 50–330 kg ha^−1^ yr^−1^ N. It is easy to establish but lacks drought tolerance [48]. Pigeon pea is an annual shrub, native to Asia and Africa. It can produce 2–25 t ha^−1^ yr^−1^ DM and up to 100 kg ha^−1^ yr^−1^ N. It is very tolerant of drought [44,47]. Lablab is a vigorous, twining annual herb, native to Africa. If used as a cover crop, it produces 2–4 t ha^−1^ yr^−1^ DM. It is drought tolerant once established [47,49]. Cowpea is a non-climbing perennial vine, native to West Africa. It can produce 3–10 t ha^−1^ yr^−1^ DM and 75–350 kg ha^−1^ yr^−1^ N. It improves soil fertility and is widely grown [44,47]. Cover crops used in this study have unique characteristics that may be useful for reducing soil degradation and improving soil fertility (Table 8).

### 3.2. Greenhouse and Mini Chamber Parameters

Duke [44] states that minimum and maximum temperatures required for perennial legume cover crops such as the ones adapted for this research are 18–28 °C. Based on such information, day/night temperatures of 30/28 °C were used for the duration of the plant growth. Two glasshouses (18 m^2^ each) were used for the growth study: one glasshouse contained ambient CO_2_ (400 μmol mol^−1^) and the second contained elevated CO_2_ (700 μmol mol^−1^). If the CO_2_ level fell below 700 μmol mol^−1^, measured by a WMA4 infrared analyzer (PP Systems, Amesbury, MA), CO_2_ was injected to reach the desired concentration. In each glasshouse, mini chambers were constructed with 2 cm (¾”) diameter PVC pipe with overall dimensions of 114 cm W × 119 cm L × 81 cm H (45” × 47” × 32”) to achieve three different levels of PPFD (100 ± 20, 200 ± 20, and 400 ± 30 µmol m^−2^ s^−1^). The different PPFD levels were achieved by covering the tops and sides of these mini chambers with plastic shade cloths: a single-ply of charcoal fiberglass window screen (New York Wire, Mt. Wolf, PA, USA) with a single-ply of 70% smoke blue sun screen fabric (Easy Gardener, Waco, TX, USA) for low PPFD, double-ply of charcoal fiberglass window screen for medium PPFD, and a single ply of 22% white shade cloth (National Tool Grinding, Inc., Erie, PA, USA) with an extra layer on the top for high PPFD. In each glasshouse, six of these shade chambers were used, two of each light intensity, to achieve the desired replication of treatments.

### 3.3. Growth Medium and Plant Growth Conditions

Growth medium consisted of a sand:perlite:peat moss (2:2:1 volume basis) mixture supplemented with essential nutrients (mg/kg) of 600 N, 600 P, 240 K, 1012 Ca, 309 Mg, 500 S, 119 Fe, 0.7 B,17.5 Mn, 7 Cu, 7 Zn and 0.35 Mo. Nutrients were applied as Osmocote 18-6-12 (The Scotts Company, Marysville, OH, USA), triple superphosphate, urea, calcium sulphate, dolomitic lime and Scott’s Micromix. The medium pH was 5.3. Cover crops seeds were planted (30 seeds/pot, 15 if big seeds) in 2.5 L plastic pots containing 2 kg of growth medium. Perennial peanut rooted seedlings were prepared by growing branch cuttings of matured plants in the greenhouse in 100% pro-mix medium. After 55 days, these rooted seedlings were transplanted to 2.5 L plastic pots containing 2 kg of growth medium. Throughout the growth cycle, the moisture level of the growth-medium was maintained near field capacity (−33 kPa). One pot without any plants was placed in each of the mini chambers to monitor the evaporative water loss.

### 3.4. Determination of Physiological Parameters

After 14 days, plants were thinned to a specific number of plants (Peanut 3, Ea-Ea 6, Mucuna 3, Pigeon pea 3, Lablab 3, Cowpea 3 plants/pot). Removed plants were used as an initial harvest.

One week before the final harvest, SPAD index values were measured with a Minolta SPAD meter (Konica Minolta Chlorophyll Meter, Model 502, Ramsey, NJ, USA). Net photosynthesis (*P_N_*, µmol (CO_2_) m^−2^ s^−1^) and its components stomatal conductance (*gs*, mol H_2_O m^−2^ s^−1^), internal CO_2_ concentration (*Ci*, cm^3^ m^−3^), transpiration rate (*E*, mmol H_2_O m^−2^ s^−1^) and vapor pressure deficit (*VPD*, kPa) were measured using a Ciras-2 Portable Photosynthesis System (PP Systems, Amesbury, MA, USA).

### 3.5. Determination of Growth Parameters

Plants were harvested after 33 days of growth. Leaves were separated from the stems and the leaf area (LA) was determined by a Li-Cor Model 3100 Leaf Area Meter (Li-Cor Inc., Lincoln, NE, USA). Leaves and stems were washed with deionized water, freeze-dried and dry weights were measured. The roots were removed from the soil, washed, blotted dry and weighed. Root lengths were determined with a Comair Root Length Scanner (Hawker de Haviland, Melbourne, Victoria, Australia). Roots were oven dried at 70 °C for 5 days and the dry root weights were recorded. Plant growth parameters were determined as follows:

Root/Shoot (R/S) Ratio = (Wr/Ws), where Wr is root dry weight and Ws is shoot dry weight, all in g plant^−1^.
Specific Leaf Area (SLA, cm^2^/g) = [Total leaf area/plant, cm^2^/Total leaf dry wt./plant, g].
Relative Growth Rate (RGR, g g^−1^day^−1^) = [ln (Wt_2_/Wt_1_)/(T_2_ − T_1_)], where Wt is total dry weight (shoot + root), T is time in days, subscripts 1 and 2 refer to initial (14 days) and final (33 days) harvests.
Net assimilation Rate (NAR, g cm^−2^ day^−1^) = [RGR/LAR], where Leaf Area Ratio (LAR, cm^2^/g) = [Total leaf area/plant, cm^2^/Shoot + Root dry wt./plant, g].

### 3.6. Determination of Water Flux (Vo) and Water Use Efficiency (WUE)

Rate of water flux (Vo, H_2_O influx/cm^2^ root s^−1^) over the growth of the crop was calculated with the formula:

Water Flux (Vo, cm^3^ plant^−1^) = [TRANS/(T_2_ − T_1_)][lnRL_2_ − lnRL_1_)/(RL_2_ − RL_1_)]}/(2πRR); where TRANS is H_2_O Transpired (g H_2_O plant^−1^), RL is root length (cm plant^−1^), T is time in seconds, subscripts 1 and 2 refer to initial and final harvests and RR is Root Radius (cm) = (RFW/RL ∗ π)^1/2^, where RFW is root fresh wt (g plant^−1^).
WUE_TOTAL_ = Total water use efficiency (g shoot Dry wt plant^−1^/g water transpired over entire growth period), where water transpired was calculated by subtracting evaporation from the total water loss for the whole experiment.
WUE_INST_ = Instantaneous water use efficiency, *P_N_*/*E* = µmol CO_2_ m^−2^ s^−1^/mmol H_2_O m^−2^ s^−1^, where *P_N_* is net photosynthesis and *E* is transpiration measured by Ciras-2.
WUE_INTR_ = Intrinsic water use efficiency *P_N_*/*gs* = (µmol CO_2_ m^−2^ s^−1^/mol H_2_O m^−2^ s^−1^), where *P_N_* is net photosynthesis and *gs* is stomatal conductance measured by Ciras-2 [50].

### 3.7. Determination of Nutrient Uptake Parameters

Dried shoot samples were ground to pass through a 0.55 mm mesh sieve. Chemical analysis of the shoot samples was conducted at the Indian River Research and Education Center, University of Florida, Fort Pierce, FL, USA). Plant tissues (0.4 g) were digested in 5 mL of concentrated 14 N HNO_3_ [51]. The concentrations of macro- (N, P, K, Ca, and Mg) and micro-elements (Cu, Fe, Mn, and Zn) in the digest solution samples were analyzed using inductively coupled plasma optical emission spectrometry (ICPOES, Ultima JY Horiba Inc., Edison, NJ, USA) following USEPA method 200.7 [52]. Total N in the plant tissue was analyzed by the combustion method using CN Analyzer (Vario MAX CN Macro Analyzer, Elementar Analysensysteme GmbH, Hanau, Germany) [53].

Nutrient uptake (U), influx (IN), transport (TR) and use efficiency (NUE) were calculated as follows:

1. Nutrient Uptake (U, mg plant^−1^) = Concentration of any given element (mg/g or µg/g) × Shoot Dry Weight (g plant^−1^).
2. Nutrient Influx (IN, pmoles cm roots^−1^ s^−1^) = [(U_2_ − U_1_)/(T_2_ − T_1_)] [(lnRL_2_ − ln RL_1_)/(RL_2_ − RL_1_)], where U refers to elemental uptake in shoot (mmole plant^−1^), T is time in seconds, RL is root length (cm plant^−1^); and subscripts 1 and 2 refer to initial and final harvest time.
3. Nutrient Transport (TR, pmoles g shoots^−1^ s^−1^) = [(U_2_ − U_1_)/(T_2_ − T_1_)] [(lnWs_2_ − ln Ws_1_)/(Ws_2_ − Ws_1_)], where U refers to elemental uptake in shoot (mmole plant^−1^), T is time in seconds, Ws is shoot dry weight and subscripts 1 and 2 refer to initial and final harvest time.
4. Nutrient Use Efficiency (NUE, mg of shoot dry weight/mg element) = [mg of Ws/mg of any given element in shoot].

### 3.8. Statistical Analysis

A split-split plot design was used, where CO_2_ treatments were main plots, PPFD levels were sub plots and cover crops species were sub-sub plots. Each experimental unit was replicated three times. Results were subjected to analysis of variance using general linear model (GLM) procedures of SAS (Ver. 9.1, SAS Institute, Cary, NC, USA). Statistical significant differences at 0.05% (*) and at 0.01% (**) probability levels for treatments and their interactions and statistical significance at LSD_0.05_ were determined.

## 4. Conclusions

The quality of PPFD under artificial shade is very different to PPFD under shade trees in the field. Depending on the characteristics of the upperstory tree canopy, different levels of blue and red light are absorbed and/or transmitted, which can affect understory cover crops growth and net photosynthesis differently from cover crops grown under artificial light. The following conclusions are based on the response of cover crops grown under artificial shade levels (PPFD).

Irrespective of levels of [CO_2_] and PPFD Mucuna recorded the highest shoot dry weight, root length and leaf area than other adapted cover crops. Therefore, from the obtained results, it can be concluded that it is possible to identify perennial legume cover crop species adaptable as cover crops in the early stages of establishment of tropical plantation crops, where, with time, the level of PPFD changes from adequate to inadequate due to increasing canopy cover of upperstory trees. Such cover crops could reduce soil erosion, leaching of nutrients and improve soil health. Interspecific differences were observed between tropical perennial legume cover crop species for growth, physiological and macro-micronutrient uptake parameters under increasing levels of [CO_2_] and PPFD. To enhance the longevity of legume cover crops in early plantation establishment it is vital to manage the canopy of upperstory main and shade trees in order to improve light levels reaching the canopy of understory cover crops.

## Figures and Tables

**Table 1:** The effect of [CO_2_] and PPFD on shoot and root growth of leguminous cover crops.

Species	PPFD (µmol m^−2^ s^−1^)	Total Dry Weight (g plant^−1^)	Root Dry Weight (g plant^−1^)	Root/Shoot Ratio	Stem Height (cm plant^−1^)	Total Root Length (cm plant^−1^)	Total Leaf Area (cm^2^ plant^−1^)	Specific Leaf Area (cm^2^ g^−1^)	Relative Growth Rate (g g^−1^d^−1^) (×10^−2^)
400 µmol CO_2_ mol^−1^									
P. Peanut	100	1.39	0.26	0.24	100	919	264.5	349.3	4.99
	200	1.94	0.35	0.22	143	1162	331.5	320.2	6.05
	400	1.81	0.53	0.41	92	1319	237.1	270.3	5.70
Ea-Ea	100	0.05	0.00	0.04	10	68	26.4	705.9	13.66
	200	0.22	0.01	0.04	15	346	78.4	490.1	18.02
	400	0.36	0.03	0.09	16	569	82.0	352.1	19.50
Mucuna	100	4.15	0.21	0.05	107	2682	1770.6	634.9	9.87
	200	4.11	0.22	0.06	95	2654	1400.8	557.7	10.00
	400	3.04	0.27	0.10	98	2486	790.3	502.0	9.11
Pigeon pea	100	0.48	0.02	0.04	34	244	147.5	455.5	7.89
	200	1.33	0.07	0.05	42	407	280.3	321.3	10.90
	400	1.58	0.09	0.06	36	776	231.3	230.8	11.27
Lab-Lab	100	1.03	0.07	0.07	79	805	396.8	609.6	8.53
	200	1.86	0.10	0.05	76	1199	628.9	492.7	10.34
	400	1.83	0.10	0.06	52	1227	448.1	328.4	10.29
Cowpea	100	1.43	0.07	0.05	67	622	425.9	527.1	9.01
	200	2.64	0.09	0.04	84	1166	610.5	440.4	10.80
	400	5.31	0.34	0.07	112	2891	736.9	290.7	12.81
700 µmol CO_2_ mol^−1^									
P. Peanut	100	1.54	0.28	0.24	146	964	338.0	414.3	5.28
	200	1.83	0.37	0.26	133	1153	322.6	346.1	5.86
	400	3.01	0.82	0.37	171	2570	334.7	252.5	7.38
Ea-Ea	100	0.07	0.00	0.04	13	97	32.7	618.4	14.37
	200	0.27	0.02	0.08	20	279	82.1	496.3	17.97
	400	0.56	0.05	0.10	20	623	109.2	333.6	19.76
Mucuna	100	4.25	0.19	0.05	106	2226	1574.5	604.3	9.77
	200	4.56	0.24	0.06	120	2734	1247.9	514.8	10.00
	400	3.49	0.23	0.07	107	2322	694.6	398.8	9.23
Pigeon pea	100	0.64	0.03	0.05	41	283	196.9	482.6	8.41
	200	1.49	0.10	0.07	56	719	271.3	327.0	10.89
	400	1.71	0.16	0.10	47	1264	229.2	242.3	11.32
Lab-Lab	100	1.69	0.09	0.05	85	1449	632.6	578.7	9.76
	200	2.10	0.14	0.07	80	1661	592.5	443.7	10.39
	400	1.78	0.12	0.07	62	1454	411.7	316.1	9.84
Cowpea	100	2.06	0.09	0.05	100	968	519.1	493.9	9.80
	200	3.62	0.13	0.04	104	1601	756.8	398.5	11.40
	400	7.48	0.59	0.08	107	3624	822.1	274.2	13.47
Significance									
CO_2_ (C)		**	**	NS	**	**	NS	**	*
PPFD (P)		**	**	**	NS	**	**	**	**
Species (S)		**	**	**	**	**	**	**	**
C × P		NS	**	NS	NS	NS	NS	NS	NS
C × S		**	*	NS	NS	**	NS	**	NS
P × S		**	**	**	NS	**	**	**	**
C × P × S		NS	*	NS	*	NS	NS	NS	NS
LSD_0.05_		1.62	0.20	0.08	57.1	1062	495.6	113.8	2.34

*, ** Significant at 0.05 and 0.01 levels of probability, respectively. NS = Not significant.

**Table 2:** The effect of [CO_2_] and PPFD on shoot and root growth of leguminous cover crops.

Species	PPFD (µmol m^−2^ s^−1^)	SPAD	NAR (×10^−4^)	*P_N_*	*gs*	*Ci*	*E*	Water Flux Vo	*WUE _TOTAL_* (×10^−3^)	*WUE_INST_*	*WUE_INTR_*
400 µmol CO_2_ mol^−1^											
P. Peanut	100	26.6	2.65	3.4	0.094	306	2.27	0.84	3.42	1.49	36.2
	200	24.4	3.54	4.6	0.045	189	1.11	1.36	2.75	4.23	108.1
	400	26.6	4.33	6.5	0.047	142	1.22	0.72	3.17	5.38	141.8
Ea-Ea	100	25.0	2.63	3.6	0.076	269	1.61	15.80	1.46	2.32	49.7
	200	29.5	5.15	6.6	0.080	206	1.62	4.69	4.06	4.05	83.9
	400	33.5	8.56	10.5	0.111	173	2.22	4.05	3.99	4.77	98.4
Mucuna	100	37.4	2.31	2.7	0.052	264	1.22	1.79	6.67	2.25	52.7
	200	36.7	2.95	3.8	0.046	21	1.06	4.57	3.27	3.53	81.9
	400	27.1	3.59	5.4	0.047	161	1.12	5.49	1.84	4.88	116.1
Pigeon pea	100	33.4	2.59	3.7	0.110	292	2.22	9.22	1.60	1.66	33.7
	200	39.7	5.16	6.8	0.134	258	2.30	6.76	2.89	3.06	52.3
	400	35.9	7.71	9.6	0.136	215	2.69	6.66	2.65	3.65	73.1
Lab-Lab	100	39.4	2.20	3.1	0.074	279	1.49	5.32	2.29	2.14	43.3
	200	34.8	3.06	4.7	0.074	229	1.50	6.70	2.51	3.37	73.0
	400	32.0	4.21	9.2	0.125	207	2.45	7.24	2.18	3.86	79.6
Cowpea	100	44.1	3.05	3.3	0.107	296	2.21	6.15	2.22	1.50	31.3
	200	42.5	4.66	6.9	0.110	234	2.16	6.74	3.18	3.25	65.3
	400	47.4	9.36	9.6	0.090	145	1.94	6.06	3.49	5.27	118.1
700 µmol CO_2_ mol^−1^											
P. Peanut	100	25.1	2.40	4.5	0.056	505	1.34	0.28	10.42	3.65	90.8
	200	26.1	3.34	8.6	0.069	432	1.65	0.75	4.31	5.46	133.9
	400	20.9	6.67	6.5	0.028	275	0.75	0.68	4.08	9.03	243.9
Ea-Ea	100	26.7	3.26	4.6	0.054	502	0.84	9.38	3.42	5.55	86.6
	200	31.4	5.92	10.1	0.079	423	0.98	7.84	2.84	10.24	128.8
	400	35.5	9.79	11.6	0.083	342	1.11	4.06	4.74	11.74	177.6
Mucuna	100	36.1	2.63	3.4	0.027	402	0.45	3.60	4.59	8.94	155.1
	200	32.8	3.71	7.1	0.077	480	1.03	3.91	3.54	7.10	96.1
	400	23.2	4.75	11.1	0.091	404	1.23	4.97	2.41	9.85	136.8
Pigeon pea	100	34.0	2.74	4.9	0.073	518	1.42	8.11	1.81	3.51	71.2
	200	37.4	5.97	8.0	0.109	513	1.32	6.03	2.63	6.08	75.4
	400	38.4	8.54	12.4	0.114	415	1.53	5.85	2.40	8.92	127.9
Lab-Lab	100	38.0	2.62	4.8	0.070	515	1.58	4.33	2.89	3.16	72.7
	200	35.0	3.69	7.7	0.089	489	1.16	5.80	2.46	6.66	87.8
	400	34.7	4.28	14.8	0.207	488	2.39	5.55	2.36	6.47	78.4
Cowpea	100	45.1	3.90	4.3	0.075	546	0.99	6.50	2.66	4.45	59.6
	200	44.5	5.46	9.6	0.149	482	1.71	6.94	3.21	6.46	90.1
	400	47.4	12.57	11.5	0.085	401	1.13	6.50	3.01	10.39	139.5
Significance											
CO_2_ (C)		NS	**	**	NS	**	**	NS	*	**	**
PPFD (P)		NS	**	**	*	**	**	NS	NS	**	**
Species (S)		**	**	**	**	**	**	**	**	**	**
C × P		NS	**	*	*	NS	*	NS	*	NS	**
C × S		NS	NS	NS	NS	NS	NS	NS	**	NS	**
P × S		**	**	**	**	**	**	**	**	**	**
C × P × S		NS	NS	NS	NS	NS	NS	NS	**	NS	NS
LSD_0.05_		9.4	2.62	5.08	120.5	155.7	1.89	8.32	8.01	5.23	107.4

*, ** Significant at 0.05 and 0.01 levels of probability, respectively. NS = Not significant. NAR = Net Assimilation Rate (g cm^−2^ d^−1^); *P_N_* = Net Photosynthesis (µmol CO_2_ m^−2^ s^−1^); *gs* = stomatal conductance (mol H_2_O m^−2^ s^−1^); *Ci* = Internal CO_2_ (cm^3^ m^−3^); *E* = Transpiration (mmol H_2_O m^−2^ s^−1^); *WUE_TOTAL_ =* Total water use efficiency (g shoot/g trans); *WUE_INST_* = Instantaneous water use efficiency (*P_N_/E*, µmol mmol^−1^); *WUE_INTR_* = Intrinsic water use efficiency (*P_N_/gs*, µmol mol^−1^).

**Table 3:** The effect of [CO_2_] and PPFD on nutrient concentration of leguminous cover crops.

Species	PPFD (µmol m^−2^ s^−1^)	N	P	K	Ca	Mg	Cu	Fe	Mn	Zn
		mg g^−1^					µg g^−1^			
400 µmol CO_2_ mol^−1^										
P. Peanut	100	26.55	9.15	29.37	22.56	4.89	4.34	46.23	85.5	57.7
	200	23.81	7.94	28.63	21.08	5.25	4.30	36.38	69.7	54.6
	400	24.07	7.96	29.37	19.08	5.13	3.86	29.82	63.7	47.5
Ea-Ea	100	44.06	6.61	26.41	8.87	2.82	13.35	39.87	425.4	49.4
	200	41.24	7.11	23.67	11.76	3.35	13.87	33.60	447.3	49.6
	400	42.81	9.65	20.19	11.00	3.33	17.26	21.80	439.1	80.1
Mucuna	100	57.75	9.95	17.61	10.34	2.26	25.38	50.29	531.8	57.2
	200	51.18	13.09	18.89	10.34	2.73	32.85	59.40	375.8	104.5
	400	49.91	14.66	19.11	9.31	2.80	37.54	54.37	314.6	140.2
Pigeon pea	100	46.98	18.20	29.25	9.05	2.17	14.38	49.38	393.9	104.3
	200	43.96	17.97	25.60	9.93	2.08	13.34	43.75	338.7	146.0
	400	45.65	16.57	23.53	8.31	2.20	16.33	72.96	342.4	126.1
Lab-Lab	100	62.27	14.11	32.14	11.63	1.74	11.10	87.80	345.5	77.5
	200	61.12	18.70	27.69	12.83	2.44	13.44	139.17	492.8	107.8
	400	64.46	20.25	27.51	11.85	2.57	16.07	139.69	561.4	124.8
Cowpea	100	62.99	7.82	37.84	16.00	3.68	7.31	71.00	677.5	104.3
	200	61.46	8.57	33.69	14.91	3.67	8.55	74.45	712.1	122.1
	400	56.34	11.85	25.73	9.30	3.80	9.26	72.22	618.3	111.5
700 µmol CO_2_ mol^−1^										
P. Peanut	100	23.36	7.75	30.41	21.98	5.15	5.76	38.26	77.5	48.1
	200	22.39	7.74	29.40	22.17	5.44	5.53	32.06	78.0	45.7
	400	14.28	6.28	25.69	21.68	5.31	5.87	31.18	74.3	40.8
Ea-Ea	100	37.24	5.17	27.33	8.58	2.85	14.41	46.00	300.1	61.9
	200	37.54	6.21	21.49	10.44	3.08	13.08	26.79	396.0	59.0
	400	39.46	7.86	18.95	9.37	3.25	12.49	18.76	377.1	46.0
Mucuna	100	47.22	11.58	19.56	10.36	2.32	30.60	50.05	455.2	61.6
	200	50.87	14.27	18.27	10.04	2.55	31.52	54.84	332.4	102.2
	400	40.56	16.28	18.36	11.07	2.98	38.89	68.01	312.0	120.4
Pigeon pea	100	47.72	18.50	35.01	10.60	2.60	18.52	64.85	456.7	126.1
	200	44.43	16.57	25.40	9.52	2.20	15.89	52.58	294.7	100.7
	400	43.60	17.88	24.01	10.14	2.81	24.37	101.73	344.2	152.3
Lab-Lab	100	56.09	14.58	28.86	13.02	1.68	13.81	109.25	391.6	112.7
	200	52.66	16.82	25.50	12.99	2.11	14.39	127.60	457.7	137.8
	400	63.26	20.48	28.71	11.09	2.44	13.02	104.33	503.2	103.6
Cowpea	100	61.80	7.61	35.15	13.21	3.26	9.35	88.05	612.1	128.8
	200	54.17	8.92	20.29	17.89	5.18	7.38	74.20	306.0	67.1
	400	48.73	12.44	24.83	12.43	4.82	10.67	155.23	886.1	161.8
Significance										
CO_2_ (C)		**	NS	*	NS	**	**	NS	NS	NS
PPFD (P)		**	**	**	**	**	**	NS	NS	**
Species (S)		**	**	**	**	**	**	**	**	**
C × P		NS	NS	**	NS	NS	*	NS	NS	NS
C × S		NS	NS	**	NS	**	**	*	NS	NS
P × S		**	**	**	*	**	**	**	**	*
C × P × S		NS	NS	**	NS	**	*	NS	**	*
LSD_0.05_		12.03	5.10	6.94	6.01	0.83	6.80	66.32	342.6	85.3

*, ** Significant at 0.05 and 0.01 levels of probability, respectively. NS = Not significant.

**Table 4:** The effect of [CO_2_] and PPFD on nutrient uptake by leguminous cover crops.

Species	PPFD (µmol m^−2^ s^−1^)	N	P	K	Ca	Mg	Cu	Fe	Mn	Zn
		mg plant^−1^					µg plant^−1^			
450 µmol CO_2_ mol^−1^										
P. Peanut	100	30.0	10.16	33.00	25.41	5.55	4.9	53.1	98	66.4
	200	37.6	12.57	45.37	33.37	8.31	6.8	57.3	109	86.5
	400	29.7	9.72	36.81	24.07	6.45	4.9	37.6	85	59.9
Ea-Ea	100	2.1	0.32	1.28	0.43	0.14	0.6	1.9	21	2.4
	200	8.8	1.54	5.07	2.54	0.72	2.9	7.2	98	11.0
	400	14.1	3.18	6.64	3.62	1.10	5.7	7.1	144	26.5
Mucuna	100	223.1	39.75	69.72	42.49	8.97	103.5	223.4	2089	237.2
	200	198.6	50.87	73.40	40.11	10.62	127.5	230.8	1459	406.0
	400	138.3	40.62	53.04	25.77	7.77	103.9	150.4	874	385.8
Pigeon pea	100	21.6	8.33	13.44	4.19	0.99	6.6	22.8	176	48.0
	200	55.1	22.62	32.18	12.52	2.63	16.8	55.4	428	184.3
	400	68.1	24.40	35.21	12.23	3.23	23.7	102.9	493	183.1
Lab-Lab	100	63.2	13.51	30.42	11.09	1.67	10.5	82.8	329	72.9
	200	106.9	33.17	48.83	22.75	4.33	23.7	247.7	880	191.6
	400	111.2	34.77	47.39	20.33	4.41	27.6	239.5	963	215.1
Cowpea	100	86.0	10.65	51.52	21.79	5.01	9.9	95.6	916	141.7
	200	155.0	21.98	85.57	38.09	9.36	21.8	191.3	1814	307.9
	400	278.3	59.19	125.17	45.89	19.10	47.7	369.6	3073	566.8
700 µmol CO_2_ mol^−1^										
P. Peanut	100	29.4	9.86	38.05	27.39	6.49	6.9	47.4	99	59.7
	200	33.0	11.30	43.19	32.64	8.03	7.9	47.0	113	69.2
	400	31.3	13.73	56.31	47.71	11.71	12.8	68.2	164	91.1
Ea-Ea	100	2.6	0.37	1.94	0.61	0.20	1.0	3.3	21	4.4
	200	9.4	1.58	5.42	2.63	0.79	3.3	6.6	101	15.7
	400	19.9	4.11	9.44	4.64	1.65	6.3	8.7	203	22.8
Mucuna	100	193.2	47.11	78.84	41.85	9.36	124.1	203.7	1864	250.3
	200	219.2	61.51	78.83	43.23	10.99	135.4	236.5	1437	437.5
	400	134.7	53.47	59.92	35.77	9.68	126.3	221.2	1024	391.3
Pigeon pea	100	29.5	11.92	22.50	6.81	1.72	12.0	42.8	306	81.3
	200	61.5	23.01	35.18	13.22	3.06	21.8	72.7	403	139.3
	400	67.6	27.76	37.17	15.75	4.37	37.9	156.9	536	235.5
Lab-Lab	100	89.9	23.35	46.11	20.85	2.69	22.1	174.0	631	182.4
	200	103.4	33.17	50.04	25.44	4.15	28.3	251.1	896	264.3
	400	103.7	34.14	46.92	18.24	3.99	21.4	171.7	866	170.5
Cowpea	100	121.7	14.99	69.26	26.00	6.41	18.4	174.4	1205	254.4
	200	188.8	31.61	71.12	61.63	18.09	25.8	264.8	1108	237.6
	400	336.1	84.13	173.89	87.67	33.08	72.6	1047.4	6126	1111.1
Significance										
CO_2_ (C)		NS	**	**	**	**	**	**	*	**
PPFD (P)		**	**	**	**	**	**	**	**	**
Species (S)		**	**	**	**	**	**	**	**	**
C × P		NS	NS	NS	NS	**	NS	**	**	*
C × S		NS	NS	NS	*	**	NS	**	**	**
P × S		**	**	**	**	**	**	**	*	**
C × P × S		NS	NS	NS	NS	**	NS	**	**	**
LSD_0.05_		79.9	24.34	45.06	32.41	5.38	41.1	257.9	1443	241.7

*, ** Significant at 0.05 and 0.01 levels of probability, respectively. NS = Not significant.

**Table 5:** The effect of [CO_2_] and PPFD on nutrient influx (pmol cm root^−1^ s^−1^) by roots of leguminous cover crops.

Species	PPFD (µmol m^−2^ s^−1^)	N	P	K	Ca	Mg	Cu	Fe	Mn	Zn
							×10^−3^			
400 µmol CO_2_ mol^−1^										
P. Peanut	100	0.69	0.13	0.33	0.25	0.09	0.03	0.33	0.68	0.39
	200	0.81	0.15	0.41	0.30	0.12	0.04	0.32	0.69	0.47
	400	0.56	0.10	0.31	0.20	0.08	0.03	0.18	0.48	0.29
Ea-Ea	100	2.61	0.18	0.56	0.18	0.10	0.17	0.59	6.45	0.63
	200	2.93	0.23	0.60	0.29	0.14	0.22	0.60	8.13	0.76
	400	3.14	0.32	0.53	0.28	0.14	0.28	0.40	8.23	1.23
Mucuna	100	5.01	0.40	0.56	0.32	0.11	0.50	1.15	11.90	1.11
	200	4.54	0.53	0.61	0.32	0.14	0.65	1.31	8.57	2.02
	400	3.27	0.45	0.46	0.21	0.10	0.56	0.88	5.31	2.01
Pigeon pea	100	3.51	0.39	0.80	0.24	0.09	0.24	0.90	7.76	1.71
	200	7.80	0.91	1.65	0.63	0.21	0.54	1.94	15.70	6.24
	400	5.72	0.64	1.06	0.37	0.16	0.46	2.34	10.90	3.36
Lab-Lab	100	4.54	0.35	0.78	0.28	0.07	0.17	1.47	6.04	1.13
	200	5.91	0.49	0.97	0.44	0.14	0.29	3.42	12.40	2.27
	400	6.05	0.48	0.92	0.39	0.14	0.33	3.26	13.40	2.50
Cowpea	100	7.37	0.60	1.61	0.67	0.25	0.19	2.05	20.50	2.66
	200	9.19	0.76	1.80	0.78	0.31	0.28	2.80	27.30	3.84
	400	8.27	0.75	1.35	0.48	0.32	0.30	2.68	23.20	3.56
700 µmol CO_2_ mol^−1^										
P. Peanut	100	0.65	0.12	0.37	0.26	0.10	0.04	0.28	0.67	0.35
	200	0.69	0.13	0.39	0.29	0.11	0.04	0.26	0.71	0.37
	400	0.42	0.10	0.33	0.27	0.11	0.05	0.25	0.67	0.31
Ea-Ea	100	2.44	0.15	0.64	0.20	0.11	0.21	0.75	5.01	0.87
	200	3.53	0.26	0.72	0.34	0.17	0.27	0.63	9.55	1.22
	400	3.74	0.34	0.64	0.31	0.18	0.26	0.43	9.42	0.92
Mucuna	100	4.78	0.54	0.72	0.37	0.13	0.69	1.26	11.90	1.35
	200	4.78	0.49	0.62	0.33	0.14	0.66	1.29	8.04	2.06
	400	3.18	0.41	0.53	0.31	0.13	0.69	1.35	6.31	2.08
Pigeon pea	100	4.27	0.46	1.15	0.34	0.14	0.37	1.46	10.90	2.49
	200	5.07	0.59	1.05	0.39	0.14	0.41	1.52	8.67	2.52
	400	3.79	0.44	0.75	0.32	0.14	0.48	2.20	7.86	2.85
Lab-Lab	100	4.23	0.38	0.78	0.34	0.07	0.23	2.04	7.57	1.83
	200	4.42	0.43	0.76	0.38	0.10	0.27	2.67	9.78	2.43
	400	4.87	0.39	0.79	0.30	0.11	0.22	1.99	10.00	1.71
Cowpea	100	7.61	0.63	1.56	0.57	0.23	0.26	2.67	19.40	3.38
	200	8.38	0.79	1.12	0.97	0.46	0.26	2.92	12.40	2.26
	400	8.16	0..86	1.51	0.74	0.47	0.39	6.50	38.30	5.84
Significance										
CO_2_ (C)		*	NS	NS	NS	**	NS	NS	NS	NS
PPFD (P)		**	**	*	**	**	**	**	NS	**
Species (S)		**	**	**	**	**	**	**	**	**
C × P		NS	NS	**	NS	NS	NS	NS	*	NS
C × S		NS	*	NS	NS	**	NS	**	NS	NS
P × S		**	**	NS	NS	**	NS	**	**	NS
C × P × S		NS	NS	*	Ns	**	NS	*	**	NS
LSD_0.05_		3.01	0.30	0.67	0.38	0.11	0.30	2.50	14.50	3.60

*, ** Significant at 0.05 and 0.01 levels of probability, respectively. NS = Not significant.

**Table 6:** The effect of [CO_2_] and PPFD on nutrient transport (pmol g shoot^−1^ s^−1^) by leguminous cover crops.

Species	PPFD (µmol m^−2^ s^−1^)	N	P	K	Ca	Mg	Cu	Fe	Mn	Zn
400 µmol CO_2_ mol^−1^										
P. Peanut	100	1263	301.9	615.4	463.6	161.6	0.06	0.60	1.25	0.73
	200	1346	339.9	679.7	489.2	197.7	0.06	0.54	1.15	0.77
	400	1165	312.5	635.5	404.1	175.3	0.05	0.37	0.97	0.61
Ea-Ea	100	5228	355.7	1124.6	368.8	192.5	0.35	1.18	12.90	1.26
	200	6351	496.3	1306.5	634.3	296.9	0.47	1.30	17.64	1.65
	400	7076	721.0	1195.5	635.2	316.8	0.63	0.90	18.51	2.84
Mucuna	100	4958	396.5	550.7	316.9	110.4	0.50	1.11	11.79	1.09
	200	4447	524.3	595.8	316.2	135.6	0.64	1.29	8.38	1.98
	400	3934	537.2	550.2	258.6	125.8	0.67	1.07	6.37	2.43
Pigeon pea	100	3443	383.3	786.7	238.1	90.1	0.24	0.89	7.44	1.68
	200	4264	497.8	898.0	340.4	115.9	0.29	1.06	8.45	3.07
	400	4564	512.2	849.8	292.0	125.8	0.36	1.81	8.71	2.71
Lab-Lab	100	5153	394.8	890.0	316.0	77.7	0.19	1.67	6.84	1.28
	200	5605	465.9	909.3	412.7	129.2	0.27	3.19	11.59	2.13
	400	5895	463.2	898.4	378.1	134.9	0.32	3.18	13.08	2.44
Cowpea	100	4948	405.3	1079.6	446.2	164.7	0.13	1.38	13.78	1.78
	200	5699	475.7	1129.6	489.0	195.6	0.18	1.73	17.04	2.45
	400	6096	552.3	998.2	352.8	237.4	0.22	1.97	17.17	2.61
700 µmol CO_2_ mol^−1^										
P. Peanut	100	1138	312.1	660.7	466.7	176.8	0.08	0.50	1.18	0.62
	200	1197	330.3	678.7	501.3	199.5	0.08	0.45	1.25	0.63
	400	857	375.3	674.7	557.8	222.8	0.09	0.52	1.37	0.64
Ea-Ea	100	4620	290.6	1216.9	372.7	203.4	0.39	1.43	9.51	1.65
	200	5731	429.6	1176.0	557.8	271.9	0.44	1.02	15.46	1.95
	400	6591	596.2	1131.2	545.1	312.2	0.46	0.77	16.21	1.64
Mucuna	100	4024	454.6	603.2	310.2	111.9	0.58	1.06	10.00	1.14
	200	4415	447.5	574.5	306.2	126.3	0.61	1.19	7.39	1.93
	400	3252	416.4	536.1	312.9	136.7	0.70	1.37	6.42	2.11
Pigeon pea	100	3676	399.7	995.9	294.1	117.1	0.33	1.27	9.33	2.15
	200	4280	493.4	884.1	323.6	121.8	0.34	1.26	7.26	2.10
	400	4324	507.4	859.1	354.5	160.4	0.54	2.53	8.76	3.26
Lab-Lab	100	4887	441.7	897.8	396.5	84.1	0.27	2.36	8.71	2.11
	200	4824	464.5	834.7	415.7	111.1	0.29	2.91	10.69	2.69
	400	5513	442.9	893.3	338.0	122.2	0.25	2.26	11.34	1.94
Cowpea	100	5226	434.0	1075.6	394.7	157.1	0.18	1.86	13.36	2.36
	200	5278	497.9	712.7	612.1	291.1	0.16	1.82	7.73	1.41
	400	5514	576.0	1012.8	495.5	314.0	0.27	4.40	25.69	3.93
Significance										
CO_2_ (C)		**	NS	NS	NS	**	*	*	NS	NS
PPFD (P)		**	**	NS	**	**	**	**	**	**
Species (S)		**	**	**	**	**	**	**	**	**
C × P		NS	**	**	NS	NS	*	NS	*	NS
C × S		NS	**	*	NS	**	**	**	NS	NS
P × S		**	**	NS	**	**	**	**	**	*
C × P × S		NS	*	NS	NS	**	**	*	**	**
LSD_0.05_		1247	109.8	278.9	216.7	61.1	0.157	1.63	8.97	1.84

*, ** Significant at 0.05 and 0.01 levels of probability, respectively. NS = Not significant.

**Table 7:** The effect of [CO_2_] and PPFD on nutrient use efficiency (NUE) of leguminous cover crops.

Species	PPFD (µmol m^−2^ s^−1^)	N	P	K	Ca	Mg	Cu	Fe	Mn	Zn
		mg Shoot mg Element^−1^					mg Shoot mg Element^−1^ (×10^4^)			
400 µmol CO_2_ mol^−1^										
P. Peanut	100	37.72	109.9	34.12	44.3	204.4	23.12	2.17	1.18	1.75
	200	42.27	126.8	34.96	47.5	190.8	23.36	2.78	1.47	1.86
	400	42.29	128.5	34.17	52.5	195.6	26.88	3.49	1.67	2.11
Ea-Ea	100	22.70	151.5	37.98	112.7	354.2	7.50	2.51	0.24	2.03
	200	24.30	140.7	42.28	85.0	298.8	7.22	2.99	0.22	2.05
	400	23.36	103.9	49.63	91.1	301.5	5.81	4.70	0.23	1.43
Mucuna	100	17.38	100.8	56.82	99.2	443.3	4.00	2.31	0.19	1.82
	200	19.79	76.4	52.96	98.3	369.7	3.06	1.70	0.28	0.96
	400	20.06	68.3	52.39	108.2	357.9	2.67	1.84	0.32	0.72
Pigeon pea	100	21.28	55.2	34.27	110.8	465.8	6.99	2.03	0.27	0.97
	200	22.77	55.7	39.10	100.9	480.3	7.54	2.30	0.30	0.78
	400	21.92	60.6	42.54	120.7	458.6	6.17	1.42	0.30	0.82
Lab-Lab	100	15.10	70.9	31.23	86.1	576.7	9.07	1.15	0.29	1.31
	200	16.58	53.7	36.12	78.3	411.4	7.45	0.73	0.21	0.94
	400	15.54	49.6	36.42	85.0	390.5	6.23	0.72	0.18	0.81
Cowpea	100	15.89	128.0	26.44	62.5	271.9	13.73	1.51	0.15	0.96
	200	16.39	117.3	29.71	67.2	272.5	11.71	1.52	0.14	0.90
	400	17.81	84.5	39.36	111.4	263.9	11.14	1.41	0.16	0.92
700 µmol CO_2_ mol^−1^										
P. Peanut	100	42.83	129.4	32.96	46.1	194.4	17.74	2.64	1.31	2.09
	200	45.49	129.3	34.10	45.2	184.3	18.31	3.12	1.33	2.28
	400	70.06	159.7	38.96	46.1	188.5	17.23	3.25	1.35	2.50
Ea-Ea	100	26.85	193.4	36.59	116.5	350.9	6.94	2.17	0.33	1.62
	200	26.65	161.5	46.53	96.1	326.4	7.67	3.75	0.25	1.75
	400	25.38	128.3	52.84	107.0	308.2	8.01	5.47	0.27	2.18
Mucuna	100	21.26	86.8	51.24	96.8	432.5	3.35	2.02	0.22	1.66
	200	19.66	70.2	54.80	100.0	392.4	3.20	1.83	0.30	1.03
	400	25.51	61.7	54.60	91.1	336.4	2.58	1.47	0.32	0.84
Pigeon pea	100	20.96	55.9	29.28	97.4	404.4	5.65	1.65	0.24	0.82
	200	22.61	60.4	39.37	105.4	454.4	6.31	1.92	0.34	1.00
	400	22.94	56.1	41.69	99.3	358.9	4.18	0.99	0.29	0.66
Lab-Lab	100	17.85	68.7	34.75	76.8	594.7	7.25	0.92	0.26	0.91
	200	19.01	59.6	39.24	77.3	475.1	6.96	0.78	0.22	0.79
	400	15.86	49.0	34.93	90.9	411.2	7.69	0.96	0.21	0.97
Cowpea	100	16.19	132.2	28.48	75.7	307.9	10.85	1.34	0.16	0.94
	200	18.48	138.9	49.97	67.4	194.3	16.63	2.45	3.36	2.09
	400	20.85	84.9	42.05	85.9	208.1	9.63	0.69	0.11	0.62
Significance										
CO_2_ (C)		**	**	**	NS	**	**	NS	NS	NS
PPFD (P)		**	**	**	**	**	NS	**	*	*
Species (S)		**	**	**	**	**	**	**	**	**
C × P		**	NS	**	NS	NS	NS	NS	*	*
C × S		**	**	**	NS	**	**	NS	**	NS
P × S		**	**	**	**	**	NS	**	**	**
C × P × S		**	NS	**	NS	NS	NS	NS	**	**
LSD_0.05_		7.91	46.0	10.03	38.7	97.1	6.98	1.60	1.63	1.04

*, ** Significant at 0.05 and 0.01 levels of probability, respectively. NS = Not significant.

**Table 8:** Common names, scientific names, growth habits, strengths and limitations of crops used *.

No ≠	Common Name	Scientific Name	Growth Habit ¥	Strengths	Limitations
1	Perennial peanut/Amendoim forrageiro	*Arachis pintoi* Krapov. & W.C. Greg	P/N	Tolerant of low fertility, Productive, High quality pasture, Good ground cover, Combines with sward grasses, Few diseases	Slow and costly to establish, Needs good moisture, somewhat tolerant of shade and can tolerate high levels of soil manganese and aluminum, Underground seeds attract rodents, Difficult to eradicate, Susceptible to root-lesion nematodes and spider mites
2	Ea-Ea/Desmodium	*Desmodium heterocarpon* (L.) DC. subsp. o*valifolium* (Prain) Ohashi	P/N	Well adapted to acid, infertile soils, Good for restoring degraded soils, Good shade tolerance	Poor drought tolerance, Susceptible to false rust (*Synchytrium desmodii*), foliar blight (*Rhizoctonia solani*) and root-knot nematodes (*Meloidogyne javanica*)
3	Mucuna aterrimum, Velvet Bean, Mucuna preta	*Mucuna pruriens* (L.) DC var *utilis* (Wall ex Wight) L.H. Bailey	A/P/C	Fast growing, easy to produce, Improves soil fertility and yield, Resistant to many pests and diseases	Constrained by low P and high acidity, Limited drought tolerance, Susceptible to waterlogging, Toxic to monogastric animals
4	Pigeon pea, Guandu fava larga	*Cajanus cajan* (L.) Millsp.	A/N	Drought and low fertility tolerant, improves soil quality	Short life annual, brittle branches, not tolerant of waterlogging
5	Lab-lab, Hyacinth bean	*Lablab purpureus* (L.) Sweet	A/P/N	Drought tolerant, improves soil fertility, high grain yields	Annual or short-lived perennial, host to bean pests,
6	Cow pea/Fejao caupi, blackeye pea	*Vigna unguiculata* (L.) Walp.	A/P/C	Multi-purpose: leaf, grain, forage; Improves soil fertility; Ease of establishment; Adaptation to a wide range of soils; Drought and heat tolerant; High seed production	Many pests and diseases of beans

* References: [4,44,45,46,47]. ≠ Seeds from: 1. Koolau Seed Supply Co., Kaneohe, HI; 2. Ms. Arenas Beatriz, CIAT, Cali, Colombia; 3 and 5. Pirai Seed Co., PiraCicaba, SP, Brazil; 4. Globo Rural Seed Co., Goiania, Go Brazil; 6. Dr. Corival, EMBRAPA Rice and Bean Center, Goiania, GO, Brazil. ¥ A = Annual, C = Climbing, N = Non Climbing; P = Perennial.

## Data Availability

Relevant data applicable to this research are within the paper.
